# Retargeting Lentiviruses via SpyCatcher-SpyTag Chemistry for Gene Delivery into Specific Cell Types

**DOI:** 10.1128/mBio.01860-17

**Published:** 2017-12-12

**Authors:** Nagarjun Kasaraneni, Ana M. Chamoun-Emanuelli, Gus Wright, Zhilei Chen

**Affiliations:** a Department of Microbial Pathogenesis and Immunology, Texas A&M University Health Science Center, College Station, Texas, USA; b Department of Veterinary Pathobiology, Texas A&M University, College Station, Texas, USA; University of Delaware

**Keywords:** antibody, breast cancer, gene therapy, redirect, specificity

## Abstract

We report a simple strategy for the creation of lentiviral vectors specific to any desired target cells. SpyTag is inserted into an engineered Sindbis virus envelope protein and displayed on the lentivirus surface to create Sindbis virus-SpyTag pseudoparticles (Sind-SpyTag-pp). The SpyTag serves as the covalent anchoring site for a target-cell-specific cell-binding protein (CBP) that is fused to a truncated SpyCatcher (SpyCatcherΔ). Target-cell-specific lentiviruses are created by mixing the Sind-SpyTag-pp and CBP-SpyCatcherΔ *in vitro*. We first used a HER2-binding designed ankyrin repeat protein (DARPin.9.26) as the model CBP. The DARPin-conjugated lentivirus transduced HER2^+^ SKOV3 cells with an infectious titer of 5.2 × 10^6^ IU/ml, >500-fold higher than the unfunctionalized “naked” virions (<10^4^ IU/ml). The ability of the DARPin-conjugated lentivirus to transduce HER2^+^ cells correlated with the surface expression level of HER2. Furthermore, these lentiviruses preferentially transduced HER2^+^ cells in cocultures containing HER2^+^ and HER2^−^ cells. To enable the use of commercially available monoclonal antibodies (MAbs) as the CBP, we developed a convenient click chemistry-based approach to conjugate MAb-derived Fab fragments to a variant SpyCatcherΔ protein containing a nonnatural amino acid, 4-azido-l-phenylalanine (AzF). Using the HER2-binding trastuzumab as a model cell-specific MAb, we created Fab-conjugated lentiviral vectors that transduced HER2^+^ SKOV3 cells with an infectious titer of 2.8 × 10^6^ IU/ml, on par with the result achieved using the DARPin-SpyCatcherΔ fusion protein. The ability to create cell-specific lentiviral vectors through chemical conjugation of a CBP should make this approach generalizable to any antibody, giving it broad utility for a wide range of research and clinical applications.

## IMPORTANCE

Lentiviral vectors hold great potential in gene therapy. However, it remains a major hurdle to robustly engineer cell-specific lentiviral vectors. This article reports a simple and effective strategy to functionalize lentiviral vectors with cell-binding proteins, thus retargeting these viruses to cells expressing the binding partner of the CBP. The CBP is genetically or chemically linked to the SpyCatcher. The SpyTag is displayed on the virion surface as a fusion to an engineered Sindbis virus envelope protein and is used as the anchorage site for SpyCatcher-linked CBP. Using this strategy, we created lentiviral vectors highly infectious toward HER2^+^ cancer cells. The ability to rapidly create cell-specific lentiviral vectors targeting a wide range of cell types should accelerate the development of custom lentiviral vectors for many research and clinical applications.

## INTRODUCTION

Lentiviral vectors can infect both dividing and nondividing cells and integrate their transgene into the host cell’s chromosome for sustained gene expression, a favorable trait for gene therapy of many chronic and malignant diseases (1, 2). Lentivirus-based gene therapy vector Kymriah (CTL019) (3), which generates CD19 receptor-specific CAR T cells, was recently approved by the FDA for treatment of a form of acute lymphoblastic leukemia (ALL) (3). Like most current lentivirus-based gene therapies, Kymriah viruses are lentiviruses pseudotyped with vesicular stomatitis virus glycoprotein pseudoparticles (VSV-Gpp) and are delivered to patient cells *ex vivo* as VSV-Gpp promiscuously transduce a wide range of cells (4) and lack the necessary specificity needed for *in vivo* application. Development of robust cell-specific lentiviral vectors suitable for *in vivo* applications remains a major hurdle in gene therapy. To date, multiple strategies have been explored to create cell-type-specific lentiviral vectors. For example, lentiviral vectors have been pseudotyped with envelope proteins from viruses that have a natural tropism for the target cell type (5–7). Not surprisingly, most of the clinically relevant cell types (e.g., HER2^+^ cancer cells) cannot be specifically targeted by natural viral envelope proteins, and as a result, this approach is extremely limited for gene therapy applications. In addition, efficient pseudotyping often requires extensive protein engineering of the cytoplasmic region of the viral envelope protein (5, 8–10), limiting the applicability of this strategy. Adapter molecules that function as bridges between the cell-binding molecule and the viral vector have achieved some success. However, the association of adapter molecules with the viral vectors is noncovalent (11, 12), limiting their shelf life. Another strategy to reengineer the specificity of lentiviruses is through genetic fusion of a cell-binding protein to a viral envelope protein (13). Using this strategy, Bender et al. created a panel of lentiviral vectors specific to different cell types (9). Unfortunately, efficient transduction requires structural cooperation or “surface compatibility” between the cell-binding protein and the viral envelope protein, limiting the types of cells accessible for gene delivery using this approach (14).

Previously, our group developed a strategy to reengineer the cell-type specificity of lentiviruses which employed a splicing-deficient DnaE intein from *Nostoc punctiforme* (Npu) (15). The splicing-deficient C-intein fragment, NpuC* (16), was inserted into an extracellular loop of an engineered binding-deficient fusion-competent (“blinded”) Sindbis virus E2 envelope protein between residues 71 and 74 (17, 18), while the N-intein fragment, NpuN, was fused to a cell-binding protein (CBP). The noncovalent interaction between the N- and C-intein fragments enabled the virions to be functionalized with the CBP and thereby be directed to the desired cell type expressing the binding partner of CBP. Unfortunately, despite the low nanomolar affinity between the two fragments of the DnaE intein, the conjugation between the virus and the CBP was observed to be unstable, likely due to the noncovalent nature of the N- and C-intein interaction, hampering the utility of these reprogrammed viral vectors for *in vivo* applications.

**FIG 1:**
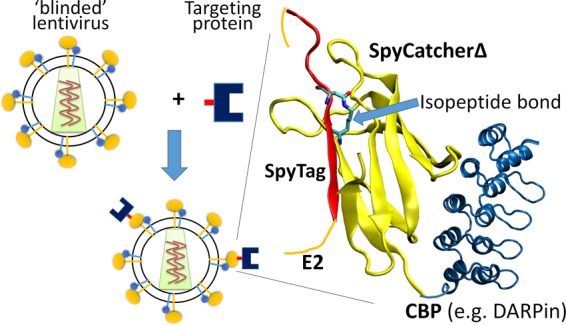
Approach for reengineering the specificity of lentiviral vectors. SpyTag (PDB ID 4MLI [red]) is inserted into a binding-deficient fusion-competent (blinded) Sindbis virus E2 envelope protein (orange) between residues 71 and 74 and used to pseudotype lentiviruses. The CBP (e.g., DARPin; PDB ID 4J7W [dark blue]) is genetically or chemically conjugated to SpyCatcherΔ (yellow). Mixing of the SpyTag-displaying lentiviral vector and CBP-SpyCatcherΔ triggers covalent functionalization of the lentivirus with the CBP through the formation of an isopeptide bond between SpyCatcherΔ and SpyTag.

In this study, we employ an isopeptide bond-forming protein-peptide pair, SpyTag and SpyCatcher, from the CnaB2 domain of the fibronectin binding protein in *Streptococcus pyogenes* (19–22) to anchor a cell surface marker-specific protein to the lentiviral surface. Although the exact function remains unknown (23), the CnaB2 domain contains a natural intramolecular isopeptide bond formed spontaneously between two adjacent residues, Lys31 and Asp117, located in two neighboring β-strands. Howarth and coworkers split the β-strand harboring Asp117 from CnaB and named it SpyTag and renamed the remaining CnaB SpyCatcher (20). SpyCatcher and SpyTag spontaneously reconstitute the fold of CnaB and rapidly form an intermolecular isopeptide bond at mild temperatures without the need for extraneous chemical reagents or catalysts (21, 24, 25). This reaction has been used in many applications, including the synthesis of an unusual nonlinear elastin-like protein polymer (24), the formation of protein hydrogels (26, 27), and the creation of multivalent antigen-presenting vaccines from virus-like particles (28). The SpyTag was used to replace NpuC* in our earlier Sindbis virus E2 envelope protein-NpuC* construct (15) to form Sind-SpyTag, and this protein was displayed on the lentivirus surface. A truncated SpyCatcher, SpyCatcherΔ, which displays significantly reduced nonspecific cell-binding effects (see Fig. S1 in the supplemental material), was genetically fused or chemically conjugated to a CBP to form CBP-SpyCatcherΔ. Coincubation of lentiviruses pseudotyped with Sind-SpyTag (Sind-SpyTag-pp) with CBP-SpyCatcherΔ yields lentiviruses covalently functionalized with the CBP, conferring these virions with specificity toward cells that express the binding partner of the conjugated CBP on their surface (Fig. 1).

10.1128/mBio.01860-17.3 FIG S1  Full-length SpyCatcher displayed significant nonspecific cell-binding activity. Download FIG S1, PDF file, 0.1 MB. Copyright © 2017 Kasaraneni et al. 2017 Kasaraneni et al. This content is distributed under the terms of the Creative Commons Attribution 4.0 International license.

Using a HER2-binding designed ankyrin repeat protein, DARPin.9.26 (29) (referred to as DARPin in this work), and a Fab fragment derived from the HER2-specific monoclonal antibody (MAb) trastuzumab (Herceptin) (30) as the model CBPs, we created lentiviral vectors that transduced HER2^+^ cells with high efficiency and specificity. DARPin was genetically fused to SpyCatcherΔ, while Fab was chemically conjugated to SpyCatcherΔ via copper-free click chemistry. Moreover, the infectivity of our SpyTag-SpyCatcherΔ virions is not negatively impacted by human serum complement, and given the covalent nature of the isopeptide bond formed between SpyCatcher and SpyTag, the association of CBP with the virions should be stable and nonreversible during circulation. These properties should render these functionalized virions appealing for *in vivo* applications.

## RESULTS

### Specificity reengineering of lentivirus with DARPin-SpyCatcherΔ.

We present a simple strategy for retargeting lentiviral vectors to deliver their genetic cargo to desired cell types. This strategy employs a peptide pair, SpyCatcher and SpyTag, for covalent functionalization of the lentiviral vectors with a cell-binding protein (CBP), thus directing the vectors to specifically transduce cells displaying the binding partner of the CBP. SpyTag was inserted into an exposed extracellular loop on Sindbis virus E2 envelope protein between residues 71 and 74 to form Sind-SpyTag (Fig. 1). DARPin.9.26 (referred to as DARPin in this work), which binds domains I to III of HER2 with a *K*_*d*_ (dissociation constant) of 1.4 nM (31), was used as the model CBP and was fused to the SpyCatcherΔ protein, which lacks 21 residues at the N terminus and 14 residues at the C terminus of the original SpyCatcher protein (22), to form DARPin-SpyCatcherΔ. The truncated SpyCatcherΔ protein was used because full-length SpyCatcher showed significant nonspecific interaction with an unknown cell surface receptor, causing significant background transduction of virions functionalized with SpyCatcher alone (Fig. S1).

**FIG 2:**
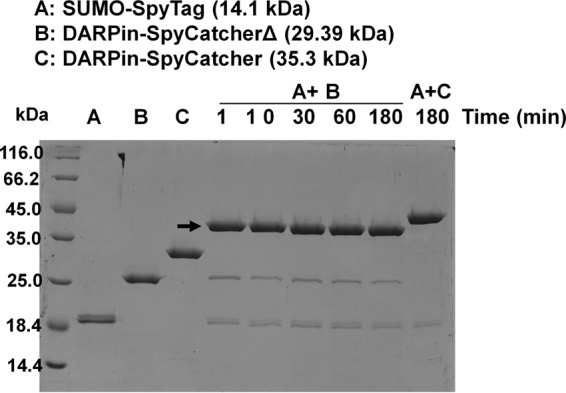
*In vitro* activity of DARPin-SpyCatcherΔ and SpyTag. One-step immobilized-metal affinity chromatography (IMAC)-purified SUMO-SpyTag (21) (20 µM) and DARPin-SpyCatcherΔ or -SpyCatcher (20 µM) were mixed in Dulbecco’s phosphate-buffered saline (DPBS) and incubated at room temperature. Samples were taken at different time points and analyzed on a 12% SDS-PAGE gel after Coomassie staining. The arrow indicates the product formed by the SpyTag-SpyCatcherΔ reaction.

We first evaluated the ability of SpyCatcherΔ to form an isopeptide bond with SpyTag. Purified DARPin-SpyCatcherΔ (29.2 kDa) was incubated with SUMO-SpyTag (14 kDa) at a 1:1 molar ratio for 1 to 180 min at room temperature and analyzed via SDS-PAGE. Formation of an isopeptide bond between SpyCatcherΔ and SUMO-SpyTag yields a new protein product of ~34 kDa. As shown in Fig. 2, >90% reaction completion was achieved within the first minute of incubation, indicating that the truncated SpyCatcherΔ retains the ability to react efficiently with SpyTag.

**FIG 3:**
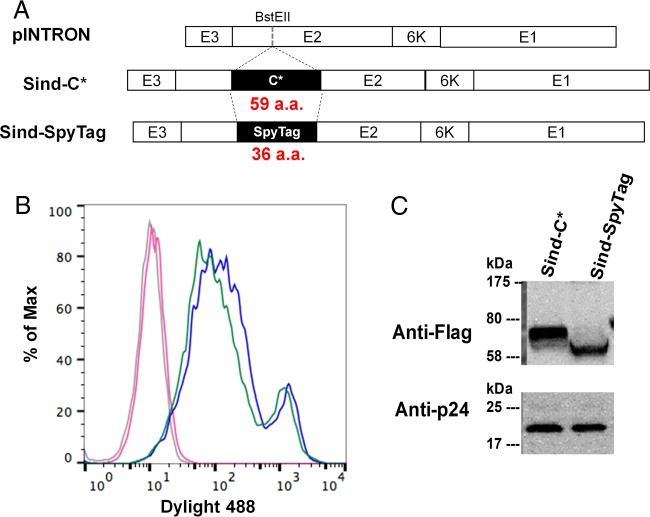
(A) Schematic representation of chimeric Sindbis envelope protein used in this study. Both Sind-C* and Sind-SpyTag contain a 3× Flag tag at the N terminus of the protein. Sind-C* and SpyTag were inserted between amino acids 71 and 74 of the Sindbis virus E2 protein. (B) Surface expression of Sind-C* (positive control [15]) and Sind-SpyTag on 293T cells detected using mouse anti-Flag and Dylight 488-conjugated goat anti-mouse antibody. Blue, Sind-C*; green, Sind-SpyTag; red, naive 293T cells stained with the same antibodies; purple, unstained naive 293T cells. (C) Incorporation of the chimeric envelope proteins into lentiviral particles. Proteins Sind(E2)-C* (~57 kDa) and Sind(E2)-SpyTag (~54 kDa) were detected using the same antibodies used in panel B or anti-p24 antibody (41).

In order for the chimeric Sind-SpyTag envelope protein to be incorporated on virions, it must first be displayed on the host mammalian cell surface. To evaluate cell surface expression of Sind-SpyTag, 293T cells were transiently transfected with plasmid encoding Sind-SpyTag or Sind-C* (15) (positive control). The presence of the chimeric proteins (Fig. 3A, which contained an N-terminal 3× Flag tag) on the cell surface, was analyzed by flow cytometry 48 h posttransfection after staining the cells with anti-Flag antibody and the appropriate Dylight 488-conjugated secondary antibody. As shown in Fig. 3B, Sind-SpyTag protein was displayed on the cell surface with similar efficiency to the positive control Sind-C*.

Next we evaluated the ability of Sind-SpyTag to be incorporated onto lentiviral vectors. Viral supernatants were concentrated 100-fold, separated on a 12% SDS-PAGE gel, and transferred to a polyvinylidene difluoride (PVDF) membrane for Western blot analysis using a mouse anti-Flag or anti-p24 primary antibody and the appropriate secondary antibody. The HIV core p24 antigen is used here as an internal loading control. As shown in Fig. 3C, Sind-SpyTag chimeric envelope protein is incorporated onto the vectors at a level comparable to the positive control Sind-C*.

**FIG 4:**
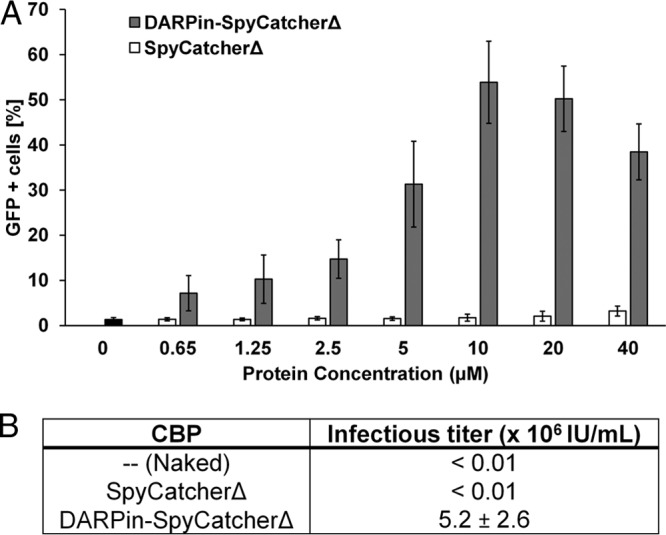
Transduction of HER2^+^ SKOV3 cells by DARPin-functionalized Sind-SpyTag-pp. (A) Bar diagram of the percentage of cells transduced with Sind-PDZ1-pp functionalized with different concentrations of DARPin-SpyCatcherΔ or SpyCatcherΔ. (B) Infectious titer of Sind-SpyTag-pp functionalized with 10 µM DARPin-SpyCatcherΔ in SKOV3 cells. Values and error bars represent the average and standard deviation, respectively, from two independent experiments.

Following the confirmation of virion incorporation of chimeric envelope protein, we determined the ability of DARPin-SpyCatcherΔ to associate with virus-displayed SpyTag and thus confer upon the virus the ability to transduce HER2^+^ cells. Unconcentrated Sind-SpyTag-displaying lentiviral pseudoparticles (Sind-SpyTag-pp, harboring a green fluorescent protein [GFP] reporter gene) were incubated with different concentrations (0 to 40 µM) of DARPin-SpyCatcherΔ or SpyCatcherΔ at room temperature for 1 h. The mixtures were diluted 50-fold in Opti-MEM medium and used to transduce the HER2^+^ ovarian cancer cell line SKOV3. As shown in Fig. 4, only background transduction was observed for naked (0 µM DARPin-SpyCatcherΔ) Sind-SpyTag-pp or those incubated with SpyCatcherΔ (<5% GFP^+^ cells), while significant amounts of GFP^+^ cells were observed in cells transduced by DARPin-SpyCatcherΔ-functionalized virions. The optimum concentration of DARPin-SpyCatcherΔ appeared to be 10 µM, which resulted in >50% GFP^+^ cells. At DARPin-SpyCatcherΔ concentrations higher than 10 μM, DARPin-SpyCatcherΔ molecules that are not bound to virions likely outcompete those incorporated onto the virions for binding to the HER2 receptors on the cell surface, resulting in reduced transduction efficiency. To determine the infectious titer of the *in vitro-*specificity-reengineered lentivirus, unconcentrated Sind-SpyTag-pp was incubated with 10 µM DARPin-SpyCatcherΔ or SpyCatcherΔ at room temperature for 1 h, and the mixtures were serially diluted and used to transduce SKOV3 cells. The infectious titer was calculated from at least four dilutions that showed linear correlation between the dilution factor and the percentage of GFP^+^ cells. Sind-SpyTag-pp functionalized with DARPin-SpyCatcherΔ efficiently transduced SKOV3 cells with an infectious titer of 5.2 × 10^6^ IU/ml, >500-fold higher than the naked Sind-SpyTag-pp (<10^4^ IU/ml) or Sind-SpyTag-pp incubated with SpyCatcherΔ (<10^4^ IU/ml) (Fig. 4B). The infectious titer of these DARPin-functionalized lentiviruses is >100-fold and >10-fold higher than the titers of previously reported specificity-reengineered lentiviruses in which DARPin proteins were displayed as fusions to an engineered measles virus envelope protein (~10^4^ IU/ml) (32) and Nipah virus envelope protein (~10^5^ IU/ml) (9), respectively.

**FIG 5:**
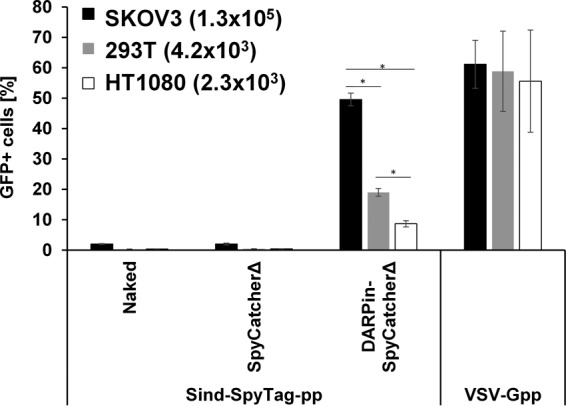
Transduction of DARPin-functionalized Sind-SpyTag-pp in cells with different surface expression levels of HER2. (The estimated number of HER2 receptors per cell is shown in parentheses [32].) The naked virions and virions functionalized with SpyCatcherΔ and VSV-Gpp were included as the controls. Values and error bars represent the average and standard deviation, respectively, from 2 independent experiments. *, *P* < 0.05 based on Student’s *t* test.

To verify that the transduction of DARPin-functionalized virions is HER2 receptor dependent, three human cell lines with different surface expression levels of HER2 were used in a transduction experiment (Fig. 5). The percentage of transduced cells in these cell lines correlated with their cell surface HER2 expression levels. The highest percentage of GFP^+^ cells was observed in cells expressing the highest level of HER2 (SKOV3, ~1.3 × 10^5^ HER2 molecules per cell [32]), and the smallest amount was observed in the cells with the lowest surface density of HER2 (HT1080, ~2.3 × 10^3^ HER2 molecules per cell [32]). The naked virus and virus functionalized with SpyCatcherΔ alone were not able to transduce any of these cells, exhibiting only background levels of GFP^+^ cells in the transduced cell population. These results confirmed that the transduction of DARPin-functionalized lentivirus is mediated by HER2 on the cell surface.

**FIG 6:**
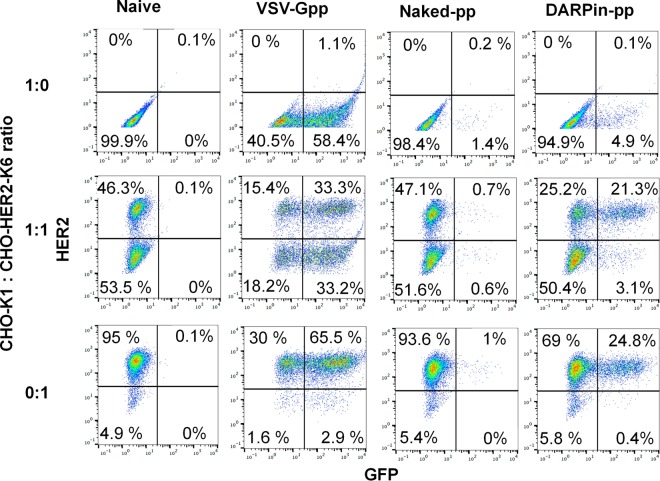
Sind-SpyTag virions loaded with HER2-binding-DARPin proteins preferentially transduce HER2-expressing cells in cocultures. Functionalized Sind-SpyTag virions selectively transduce HER2^+^ cells in coculture. CHO-K1 (HER2-negative) and CHO-HER2-K6 (HER2-high) cells, either in isolation or at a 1:1 ratio, were transduced with DARPin-functionalized Sind-SpyTag-pp, Sind-SpyTag-pp, or VSV-Gpp at 37°C for 3 h. Forty-eight hours posttransduction, cells were analyzed for GFP and HER2 expression by flow cytometry. Representative flow cytometry plots of four independent experiments are shown.

To assess the ability of DARPin-functionalized virions to selectively transduce HER2^+^ cells in a mixed cell population, we performed a coculture transduction experiment using CHO-HER2-K6, a high-HER2-expressing cell line with an estimated ~2.1 × 10^5^ HER2 molecules per cell (32), and CHO-K1 (HER2-negative) cells. Lentivirus pseudotyped with vesicular stomatitis virus (VSV-Gpp), which enters cells via the low-density lipoprotein (LDL) receptor (4) present on both cells, was used as a positive-control lentivirus. CHO-K1 and CHO-HER2-K6 cells, either in isolation or as a 1:1 mixture, were inoculated with VSV-Gpp or DARPin-functionalized Sind-SpyTag-pp. Forty-eight hours later, these cells were analyzed for HER2 expression level and lentiviral transduction (GFP fluorescence) via flow cytometry (Fig. 6). VSV-Gpp transduced CHO-K1 and CHO-HER2-K6 cells with a similarly efficiency, while the naked Sind-SpyTag-pp poorly transduced both cell lines. In contrast, DARPin-displaying Sind-SpyTag-pp showed strong preference for transducing CHO-HER2-K6 cells compared to the HER2-negative CHO-K1 cells. The ability of DARPin-loaded virus to selectively transduce the target cell type points to the potential of this strategy to produce virions capable of delivering genetic cargo specifically to target cells in an *in vivo* setting.

### Specificity reengineering of lentivirus with MAb-derived CBP.

Antibodies have played a significant role in biotechnology and modern medicine, and a large array of monoclonal antibodies (MAbs) have been developed with ultrahigh affinity and specificity toward diverse targets/receptors. Many MAbs are currently used in the clinic or are in the pipeline for therapeutic applications (33). Unfortunately, to date the advancement in MAb technology has not translated into the creation of target-specific lentiviral vectors for *in vivo* gene therapy applications, in part due to the lack of an efficient strategy for stable attachment of MAbs to the lentiviral surface. Previously, Irvine Chen’s group exploited protein A to noncovalently anchor MAbs to the lentiviral surface (17, 18, 34, 35). In that strategy, the antibody-Fc domain-specific ZZ domain from protein A was inserted into a binding-deficient, fusion-competent Sindbis virus envelope protein, enabling MAbs to be conjugated onto ZZ-displaying lentiviruses, thus redirecting these vectors to cells displaying the binding partner of the conjugated MAb. Unfortunately, the reversible interaction between the ZZ domain and the Fc of the antibody makes this strategy unsuitable for use in immunocompetent individuals, in which the conjugated antibodies are vulnerable to being displaced by serum immunoglobulins.

**FIG 7:**
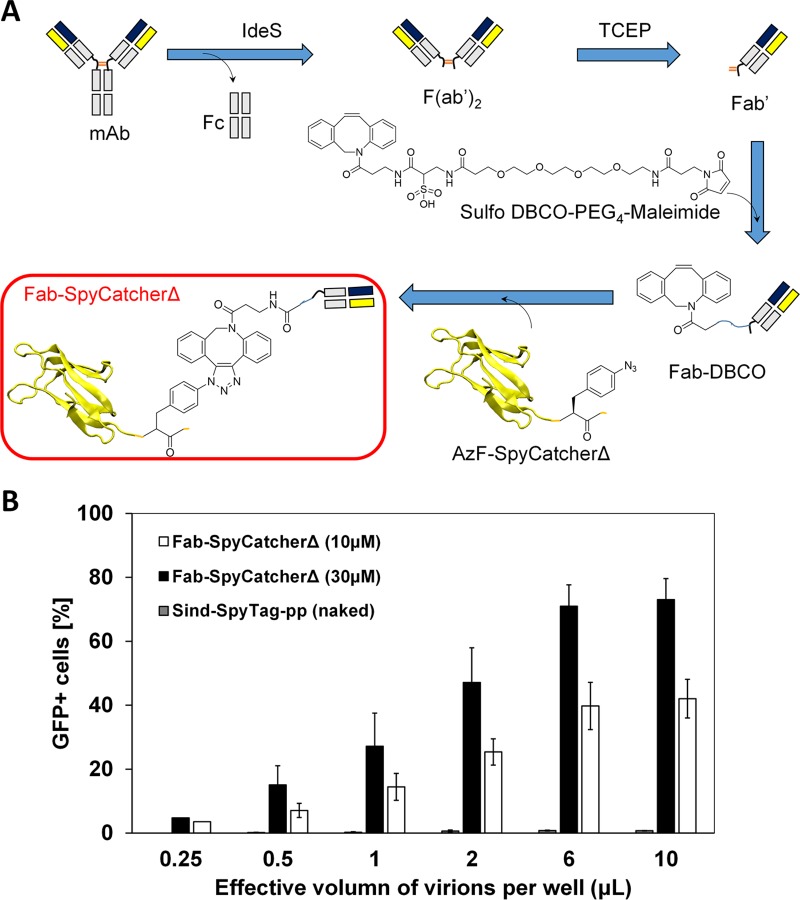
Retarget lentiviral vectors with MAb-derived CBP. (A) Scheme for the creation of Fab-SpyCatcherΔ via copper-free click chemistry. (B) Transduction of HER2^+^ SKOV3 cells by lentivirus functionalized with trastuzumab-derived Fab-SpyCatcherΔ. The functionalized Sind-SpyTag-pp were serially diluted before being used for transduction. Values and error bars represent the average and standard deviation, respectively, from two independent experiments.

To facilitate the use of commercial MAbs for the creation of cell-specific lentiviral vectors, we developed a copper-free click chemistry-mediated conjugation strategy (Fig. 7A). HER2-specific trastuzumab (30) was used as a model MAb. We first digested trastuzumab with IdeS protease (containing a His tag), which cleaves IgG molecules below the hinge region to yield F(ab′)_2_ and Fc fragments (36). The Fc and IdeS were removed using magnetic protein A beads and Ni-nitrilotriacetic acid (NTA) beads, respectively, and the resulting F(ab′)_2_ was mildly reduced to Fab′ using tris(2-carboxyethyl) phosphine (TCEP). Fab′ was then reacted with linker sulfo-dibenzocyclooctyl-polyethylene glycol-maleimide (sulfo-DBCO-PEG_4_-maleimide) to form Fab-DBCO. An unnatural amino acid, *p-*acetyl phenylalanine (AzF), was recombinantly incorporated into SpyCatcherΔ at the N terminus to form AzF-SpyCatcherΔ, which was reacted with Fab-DBCO to form Fab-SpyCatcherΔ. To evaluate the efficiency of this click chemistry-mediated specificity-reengineering strategy, unconcentrated Sind-SpyTag-pp were incubated with Fab-SpyCatcherΔ (10 µM and 30 µM) at room temperature for 1 h, and the mixture was serially diluted and used to transduce SKOV3 cells (Fig. 7B). Naked Sind-SpyTag-pp was not able to transduce SKOV3 cells. In contrast, Fab-SpyCatcherΔ-functionalized Sind-SpyTag-pp efficiently transduced SKOV3 cells with calculated infectious titers of (2.8 ± 0.5) × 10^6^ IU/ml and (7.6 ± 1.8) × 10^6^ IU/ml for virions functionalized with 10 µM and 30 µM Fab-SpyCatcherΔ, respectively. These infectious titers are similar to that of virions functionalized with 10 µM DARPin-SpyCatcherΔ (Fig. 4B). This result demonstrated our ability to direct lentiviral vectors to deliver their genetic cargo to specific cell types using MAbs as the CBPs.

### Lentiviruses functionalized via SpyCatcher-SpyTag are resistant to human serum complement.

**FIG 8:**
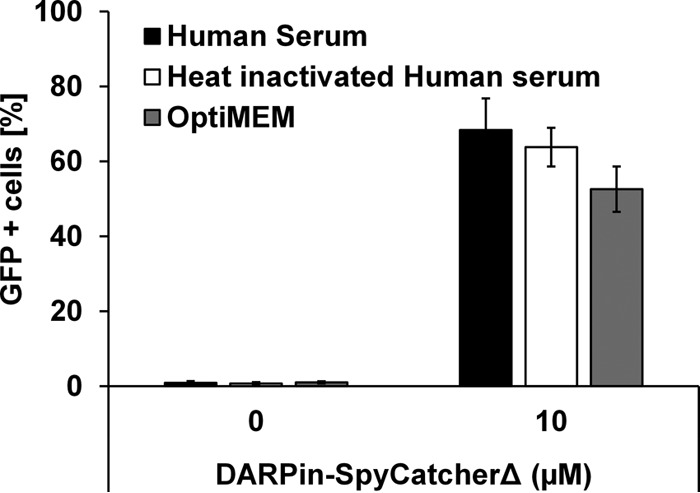
Lentiviruses surface modified via SpyCatcher-SpyTag are not inactivated by human complement. Sind-SpyTag-pp functionalized with DARPin-SpyCatcherΔ were incubated with an equal volume of untreated human serum, heat-inactivated human serum or Opti-MEM medium at 37°C for 1 h and then diluted 50-fold with Opti-MEM medium and used to transduce SKOV3 cells. The percentage of GFP^+^ cells was analyzed via flow cytometry 48 h posttransduction. Values and error bars represent the average and standard deviation, respectively, from at least three independent experiments. The difference in transduction efficiencies between samples incubated with the human serum and Opti-MEM medium is not statistically significant, with *P* = 0.15 based on Student’s *t* test.

To be effective *in vivo*, gene therapy vectors need to remain active even after exposure to the human serum complements, which attack pathogens via multiple pathways (37). To assess the ability of our specificity-reengineered lentiviral vectors to resist serum complement, DARPin-SpyCatcherΔ-functionalized Sind-SpyTag-pp were mixed with an equal volume of untreated or complement-inactivated (heat-inactivated) human serum, and the mixtures were incubated at 37°C for 1 h, diluted 50-fold in Opti-MEM medium, and used to transduce SKOV3 cells. Similar infectivities were observed in virions incubated with untreated and complement-inactivated human serum (Fig. 8), indicating that lentiviral vectors surface modified via SpyCatcher-SpyTag chemistry are compatible with human serum complement. This result is consistent with the observation that the Sindbis virus envelope proteins are resistant to human serum complement (35). This low serum sensitivity is in contrast to lentiviruses retargeted through (i) display of a CBP as fusion to the measles virus envelope protein (32, 38, 39) (relevant mainly in measles virus vaccine-naive populations) and (ii) conjugation of cell surface receptor-specific antibodies via noncovalent interaction between the antibody Fc domain and the Fc domain-specific ZZ domain from protein A (17, 18, 34, 35). In the latter method of specificity reengineering, the displayed antibodies are at risk of being displaced by circulating serum immunoglobulins in immunocompetent individuals (40).

## DISCUSSION

The progress of gene therapy has been hampered by the absence of a lentiviral vector that can be robustly and reproducibly functionalized with a cell-homing functionality to mediate specific delivery of therapeutic genes to diseased cells. Existing strategies to reengineer the cell-type specificity of viral vectors have suffered from challenges, including (i) difficulties in achieving a reproducibly high virion incorporation level (for example, when attempting to fuse a cell receptor-binding protein to viral envelope protein [13]), (ii) a lack of *in vivo* compatibility, as seen in approaches employing protein A for anchoring cell-specific antibodies (17, 18) and noncovalent bridge proteins for linking a cell-binding protein with a protein expressed on the lentiviral surface (15), and (iii) a very limited repertoire of cell types that can be targeted (e.g., when pseudotyping the lentiviral vectors with envelope proteins from viruses that have a natural tropism for the cell type of interest [5–7]).

In this study, we describe a simple approach to retarget lentiviral vectors that has the potential to greatly expand the repertoire of cell types that can be specifically targeted by lentiviral vectors. Specifically, we employed an isopeptide bond-forming protein-peptide pair—the N-terminal fragment (Spycatcher) and C-terminal fragment (SpyTag) of the collagen adhesion domain (CnaB2) from the fibronectin binding protein (FbaB) in *Streptococcus pyogenes* (19–22)—to robustly conjugate a CBP to the lentiviral surface, thus yielding stable cell-type-specific lentiviral vectors. The SpyTag is inserted into a binding-deficient and fusion-competent Sindbis virus envelope protein and displayed on the surface of pseudotyped lentiviral particles (Sind-SpyTag-pp). The surface-displayed SpyTag is used as the anchorage site for a cell-binding protein (CBP) associated with SpyCatcher. Entry of enveloped viruses into cells requires two major steps, each mediated by a different viral protein or protein domain: (i) virus-cell attachment and (ii) fusion of the viral and cellular membranes. For many viruses, abolishment of viral attachment through mutation/deletion of the attachment function does not impair the fusion function. Viruses with an abolished wild-type attachment function can potentially be retargeted to new cell types through the incorporation of cell-specific CBPs. Using a HER2-binding DARPin as our model CBP, we first created the fusion protein DARPin-SpyCatcherΔ. SpyCatcherΔ is a truncated version of SpyCatcher that lacks 21 and 14 residues at the N- and C-termini, respectively, and was used in this work because the full-length SpyCatcher was found to interact with an undefined receptor on SKOV3 cells (Fig. S1). SpyCatcherΔ was able to efficiently form an isopeptide bond with SpyTag (Fig. 2) but exhibited significantly reduced ability to nonspecifically interact with SKOV3 cells. Unconcentrated supernatant containing Sind-SpyTag-pp functionalized with DARPin-SpyCatcherΔ efficiently transduced HER2^+^ SKOV3 cells with an infectious titer of 5.2 × 10^6^ IU/ml, which is >500-fold higher than the naked Sind-SpyTag-pp (<10^4^ IU/ml) or Sind-SpyTag-pp functionalized with only SpyCatcherΔ (<10^4^ IU/ml).

A slightly higher percentage of GFP^+^ cells was observed in HER2^−^ CHO-K1 cells transduced by DARPin-SpyCatcherΔ-functionalized virions (4.9%) than the naked virions (1.4%) (Fig. 7). Since the DARPin used in this study is highly specific to HER2 and has previously been used to create HER2-specific pseudotyped measles virus (32) and Nipah virus (9) with very low background transduction of nontarget cells, we suspect that the slightly higher transduction of the CHO-K1 cells by DARPin-functionalized virions is likely due to interaction between SpyCatcherΔ and another undefined cell surface receptor on the hamster-derived CHO-K1 cells. Ongoing work aims to further reduce the background transduction of nontargeting cells.

To expand the repertoire of cell types that can be specifically transduced, a click chemistry-based strategy was developed to redirect lentiviruses to specific cell types using the Fab fragments of receptor-specific MAbs. Using the Fab fragments derived from the HER2-specific MAb trastuzumab as a model CBP, we demonstrated that Sind-SpyTag-pp could be redirected to HER2-displaying SKOV3 cells, yielding a high infectious titer of 2.8 × 10^6^ IU/ml. The ability to covalently conjugate MAb-derived CBP to lentiviruses opens the door to the potential immediate use of a plethora of existing cell receptor-specific MAbs for the creation of cell-type-specific lentiviruses, greatly expanding the cell types that can be specifically targeted for lentiviral gene delivery.

In summary, this work reports a novel *in vitro* method for functionalizing the surface of lentiviral vectors that exploits an isopeptide bond-forming protein-peptide pair: SpyTag-SpyTag. This approach should facilitate the creation of lentiviral vectors that are able to specifically deliver their genetic cargo to a wide range of different cell types, greatly broadening the potential of lentiviral vectors as gene delivery vehicles for functional genomics and gene therapy applications.

## MATERIALS AND METHODS

### Infection assays.

Unconcentrated supernatants containing Sind-SpyTag-pp (harboring a GFP reporter gene) were incubated with the indicated concentrations of purified DARPin-SpyCatcherΔ, SpyCatcherΔ, or Fab-SpyCatcherΔ at room temperature for 1 h to promote incorporation of the SpyCatcher constructs onto the virion surface. The mixture was then diluted 50-fold in Opti-MEM medium and used to transduce cells in 48-well plates (3.3 × 10^4^ cells/well) via spinoculation for 1 h at 300 × *g* and 25°C followed by incubation at 37°C for 2 h. The cells were washed to remove unbound viruses and incubated at 37°C in 5% CO_2_. Positive-control lentiviruses were pseudotyped with vesicular stomatitis virus glycoprotein (VSV-G) and contained the same GFP reporter gene. VSV-Gpp were diluted 50-fold in Opti-MEM medium before use. The percentage of transduced cells (GFP^+^) in each well was quantified 48 h later using a BD FACScan flow cytometer (BD Biosciences; San Jose, CA). For virus titer determination, functionalized virions were serially diluted before being used for transduction. The number of infectious units per milliliter was calculated from at least three dilutions that showed a linear correlation between the dilution factor and the percentage of GFP-positive cells using the equation MOI = −ln[1 − (% of GFP^+^ cells/100)], where MOI is the multiplicity of infection.

For the coculture transduction experiment, the cells were stained with anti-HER2-phycoerythrin (PE) (BD Biosciences; San Jose, CA) before being analyzed by flow cytometry.

### Conjugation of Fab to AzF-SpyCatcherΔ.

Trastuzumab (1 mg/ml in phosphate-buffered saline [PBS]) was first digested with the enzyme IdeS (containing a 6× His tag at 1% [wt/wt]) at 37°C for 1 h to convert MAb to F(ab′)_2_. The IdeS and the generated Fc fragments were removed via exposure to Ni-NTA agarose beads (Qiagen) and protein A-coated magnetic beads (Pierce), respectively. The resulting F(ab′)_2_ fragments were reduced to F(ab′) by incubation with 5 mM tris(2-carboxyethyl) phosphine (TCEP) at 37°C for 30 min, desalted using a Bio-Spin 6 column to remove TCEP, and then reacted with the linker sulfo-DBCO-PEG_4_-maleimide (10 mM; Click Chemistry Tools) in PBS at 4°C for 14 h to form Fab-DBCO. Excess unreacted linker molecules were removed by passage of the reaction mixture through another Bio-Spin 6 column. Fab-DBCO was subsequently incubated with purified AzF-SpyCatcherΔ at a 1:8 molar ratio for 16 h at room temperature to produce T-F(ab′)-SpyCatcher. Excess unreacted AzF-SpyCatcherΔ (13 kDa) was removed using a Bio-Spin 30 column. The concentration of the Fab-SpyCatcherΔ was determined using the bicinchoninic acid (BCA) assay kit (Thermo Fisher Scientific, Waltham, MA).

Additional materials and methods are provided in Text S1 in the supplemental material. The protein sequences for all constructs are provided in Text S2 in the supplemental material.

10.1128/mBio.01860-17.1 TEXT S1  Supplementary methods. Download TEXT S1, PDF file, 0.1 MB. Copyright © 2017 Kasaraneni et al. 2017 Kasaraneni et al. This content is distributed under the terms of the Creative Commons Attribution 4.0 International license.

10.1128/mBio.01860-17.2 TEXT S2  Protein sequences. Download TEXT S2, PDF file, 0.1 MB. Copyright © 2017 Kasaraneni et al. 2017 Kasaraneni et al. This content is distributed under the terms of the Creative Commons Attribution 4.0 International license.

10.1128/mBio.01860-17.4 FIG S2  Unnatural amino acid AzF is successfully incorporated into SpyCatcherΔ. Download FIG S2, PDF file, 0.1 MB. Copyright © 2017 Kasaraneni et al. 2017 Kasaraneni et al. This content is distributed under the terms of the Creative Commons Attribution 4.0 International license.
