# Supplementary material for: Retargeting Lentiviruses via SpyCatcher-SpyTag Chemistry for Gene Delivery into Specific Cell Types
Source: mBio. 2017 Dec 12;8(6):e01860-17. doi: 10.1128/mBio.01860-17 (PMC5727413; doi:10.1128/mBio.01860-17)
Supplement: FIG S1 [file mbo006173638sf1.pdf]

S3. Supplementary Figures.

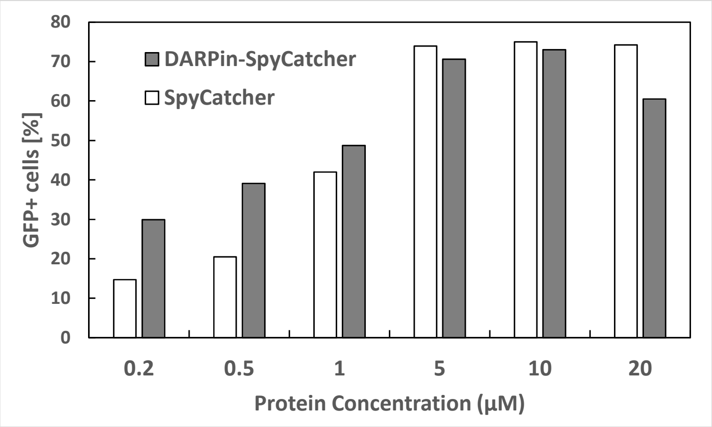

Figure S1. Full-length SpyCatcher displayed significant non-specific cell-binding activity. Sind-SpyTag-pp was incubated with increasing concentrations of DARPin-SpyCatcher (DARPin.9.26) or full-length SpyCatcher at room temperature for 1 hr, and used to transduce SKOV3 cells. Significant transduction was observed for virions incubated with SpyCatcher alone, indicating that SpyCatcher likely binds non-specifically to a cellular receptor on SKOV3 cells. This is in contrast to data presented in Figure 4, in which only DARPin-SpyCatcherΔ functionalized virions were able to efficiently transduce SKOV3 cells.
