# Supplementary material for: Retargeting Lentiviruses via SpyCatcher-SpyTag Chemistry for Gene Delivery into Specific Cell Types
Source: mBio. 2017 Dec 12;8(6):e01860-17. doi: 10.1128/mBio.01860-17 (PMC5727413; doi:10.1128/mBio.01860-17)
Supplement: TEXT S1 [file mbo006173638s1.pdf]

S1. Supplementary Materials and Methods:

Cells and chemicals

HEK 293T cells were purchased from Life Technology (Carlsbad, CA). SKOV3, HT1080, Chinese hamster ovary (CHO-K1) cells and selected clones were kindly provided by Christian Buchholz (Paul-Ehrlich Institut; Langen, Germany)[24]. Unless otherwise stated, all cell lines were cultured in Dulbecco's Modified Eagle's medium (DMEM) containing 4,500 mg/liter glucose, 4.0mML-glutamine, and 110 mg/liter sodium pyruvate (Thermo Scientific HyClone, Logan, UT) supplemented with 10% fetal bovine serum (Atlanta Biologicals, Lawrenceville, GA) and 1X non-essential amino acids (Thermo Scientific HyClone). For $HER2^+$ clone CHO-HER2-K6, the growth media was supplemented with 1.2 mg/mL of the antibiotic G418. Dulbecco's phosphate-buffered saline (DPBS) was purchased from Thermo Scientific HyClone (Logan, UT). Trastuzumab and IdeS protease were graciously provided by Prof. Zhiqiang An at the University of Texas Health Science Center.

Plasmids

Plasmids encoding HIV Gag-Pol and vesicular stomatitis virus glycoprotein (VSV-G) were kindly provided by Charles Rice (Rockefeller University, NY)[25]. pDEST14-SpyCatcher[19] was purchased from Addgene. SUMO-SpyTag was provided by Mark Howarth (University of Oxford). The SpyCatcherΔ lacks 21 residues at the N-terminus and 14 residues at the C-terminus. All protein sequences are provided in the Supplementary Materials.

Protein expression and purification

DARPin.9.26-SpyCatcherΔ (also referred as DARPin-SpyCatcherΔ) and SpyCatcherΔ were expressed in *Escherichia coli* BL21 (DE3) cells and purified via one-step IMAC as described previously[13]. The AzF-SpyCatcherΔ, harboring a non-natural amino acid – 4-azido-L-phenylalanine (AzF) at the N-terminus, was expressed using an evolved pyrrolysyl-tRNA synthetase (PylRS)-$tRNA^{Pyl}$ (pylT) pair as described previously[26, 27]. Briefly, the BL21 (DE3) cells were co-transformed with pEVOL-AzFRS[27, 28] and the AzF-SpyCatcherΔ, and plated on LB-agar plates containing 100 µg/ml of ampicillin and 34 µg/ml of chloramphenicol. The next day, the cells were grown at 37°C to an OD_{600} of ~0.6 and protein expression was induced by the addition of 0.2 mM isopropyl β-D-1-thiogalactopyranoside (IPTG), 0.02% arabinose and 5 mM 4-azido-L-phenylalanine (VWR, Radnor, PA). The cells were incubated with shaking at 18°C for 15 h following induction. Protein purification was carried out via one-step IMAC. Purified protein was concentrated, dialyzed against PBS (pH 7.4) using ultra-filtration spin columns (MWCO 10 kDa, Amicon Ultra, Millipore; Billerica, MA) and stored at -80°C until use.

To confirm the incorporation of AzF, purified AzF-SpyCatcher was reacted with DBCO-PEG4-TAMRA dye (Sigma-Aldrich, St.Louis, MO) at a 1:50 molar ratio for 1 h at 22°C in dark. The product was resolved by SDS-PAGE and visualized first under UV light and later under white light after Coomassie Brilliant Blue staining.

In vitro activity of DARPin-SpyCatcherΔ

Purified DARPin-SpyCatcherΔ (20 µM) was incubated with SUMO-SpyTag (20 µM) in DPBS at room temperature for 1-180 minutes. Aliquots were removed at different times, immediately frozen in liquid N2 and stored at -20°C. Immediately before analysis, an equal volume of 2xSDS loading buffer (0.5 M Tris-HCl, pH 6.8, 20%

glycerol, 10% w/v SDS, 0.1% w/v bromophenol blue, 2% β-mercaptoethanol) was added to each sample. The samples were denatured at 95°C for 5 minutes and loaded onto 12% SDS-PAGE gels.

Cell-surface expression of chimeric viral envelope protein

To assess the cell-surface expression level of chimeric envelope protein Sind-SpyTag (containing an N-terminus 3xFlag tag, Supplementary Material), HEK 293T cells were transiently transfected with a plasmid encoding Sind-SpyTag or Sind-C*[13] (positive control) using Trans IT reagent (Mirus Bio LLC; Madison, WI) as per the manufacturer's protocol. Forty-eight hours post transfection, cells were harvested, washed and stained with mouse anti-Flag (Genscript; Piscataway, NJ) and Dylight 488 goat anti-mouse (Jackson ImmunoResearch Laboratories, Inc; West Grove, PA) antibodies, and analyzed by flow cytometry.

Lentivirus production

Pseudotyped lentiviruses were produced by transfecting 293T cells with plasmids encoding 1) HIV gag-pol[25], 2) pTRIP-eGFP[13] and 3) the appropriate chimeric envelope protein at a 1:1:4 weight ratio using the TransIT reagent. The supernatants containing the pseudotyped lentiviruses were collected 48 h later, filtered (0.22 µm pore size) and stored at -80°C in aliquots.

Western Blot analysis of incorporated chimeric envelope proteins on virions

Lentiviruses pseudotyped with Sind-SpyTag or Sind-C* chimeric envelope proteins were harvested, concentrated by ultracentrifugation (90 min; 40000 xg; 4°C), resuspended in DPBS at 1/100th of their original volume and mixed with an equal volume of 2X SDS loading buffer. Samples were incubated for 5 min at 95 °C, resolved on a 12% SDS-PAGE gel and electrotransferred onto a polyvinylidene difluoride (PVDF) transfer membrane (Pall Corporation; Pensacola, FL). Western blot analysis was performed using mouse anti HIV-1 p24 (NIH AIDS Reagent Program, Division of AIDS, NIAID, NIH: Monoclonal Antibody to HIV-1 p24 (No. 71-31) from Dr. Susan Zolla-Pazner)[29] or mouse anti-Flag (Genscript; Piscataway, NJ) and horseradish peroxidase-conjugated goat anti-mouse (Jackson ImmunoResearch; West Grove, PA) antibodies. Protein bands were visualized by chemiluminescence using a ChemiDoc-It imager (UVP, LLC; Upland, CA).

Serum sensitivity assay

To determine the effect of serum complement on virus infectivity, undiluted Sind-SpyTag-pp was incubated with DARPin-SpyCatcherΔ at room temperature for 1 h, and the mixture was incubated with an equal volume of untreated or heat-inactivated human AB serum (Corning) at 37°C for 1 h. The serum-treated mixture was diluted 50-fold in OptiMEM medium and used to transduce SKOV3 cells as described above.
