# Supplementary material for: Retargeting Lentiviruses via SpyCatcher-SpyTag Chemistry for Gene Delivery into Specific Cell Types
Source: mBio. 2017 Dec 12;8(6):e01860-17. doi: 10.1128/mBio.01860-17 (PMC5727413; doi:10.1128/mBio.01860-17)
Supplement: TEXT S2 [file mbo006173638s2.pdf]

S2. Protein sequences:

Sind-SpyTag

Sind-SpyTag was constructed by inserting a gene encoding SpyTag into the BstEII restriction sites (between residues 71 and 73 in E2 envelope protein) in 2.2-ZZ [1,2].

(BstEII-*3xFlag*-**SpyTag**-BstEII)
VT*DYKDHDGDYKDHDIDYKDDDDK***AHIVMVDAYKPT**KGVT

Sind-C*

(BstEII-*3xFlag*-**C***-BstEII)
VT*DYKDHDGDYKDHDIDYKDDDD*K**MIKIATRKYLGKQNVYGIGVERDHNFALKNGFIASA**GVT

DARPin-SpyCatcherΔ

HER2-binding DARPins were provided by Andreas Plückthun (University of Zurich; Zurich, Switzerland)[3,4]. DARPin.9.26-SpyCatcer was constructed by overlap extension PCR and inserted into pET15b vector between NdeI and XhoI sites.

(Myc-tag-DARPin.9.26-*Linker*-**SpyCatcherΔ**-6His)
MEQKLISEEDLGSDLGKKLLEAARAGQDDEVRILMANGADVNAKDFYGITPLHLAAAYGH
LEIVEVLLKHGADVNAHDWNGWTPLHLAAKYGHLEIVEVLLKHGADVNAIDNAGKTPLHL
AAAHGHLEIVEVLLKYGADVNAQDKFGKTAFDISIDNGNEDLAEILQEAC*GGGGSGGGGS*
AS**SGDSATHIKFSKRDEDGKELAGATMELRDSSGKTISTWISDGQVKDFYLYPGKYTFVE**
TAAPDGYEVATAITFTVNEQGQVTVNGLEHHHHHH

AzF-SpyCatcherΔ

SpyCatcherΔ was PCR amplified and inserted into pET15b vector between NdeI and XhoI site. AzF is encoded by TAG stop codon

(6His-**AzF**-*Linker*-SpyCatcherΔ)
MGSSHHHHHHSSGLVPRGSHNYHMWELQQS**AzF***GG*SGDSATHIKFSKRDEDGKELAGATMEL
RDSSGKTISTWISDGQVKDFYLYPGKYTFVETAAPDGYEVATAITFTVNEQGQVTVNG

SUMO-SpyTag

(6His-SUMO-*Linker*-**SpyTag**)
MGSSHHHHHHGSDSEVNQEAKPEVKPEVKPETHINLKVSDGSSEIFFKIKKTTPLRRLME
AFAKRQGKEMDSLRFLYDGIRIQADQTPEDLDMEDNDIIEAHREQI*GGG***AHIVMVDAYKP**
TKGY
