# Supplementary material for: Retargeting Lentiviruses via SpyCatcher-SpyTag Chemistry for Gene Delivery into Specific Cell Types
Source: mBio. 2017 Dec 12;8(6):e01860-17. doi: 10.1128/mBio.01860-17 (PMC5727413; doi:10.1128/mBio.01860-17)
Supplement: FIG S2 [file mbo006173638sf2.pdf]

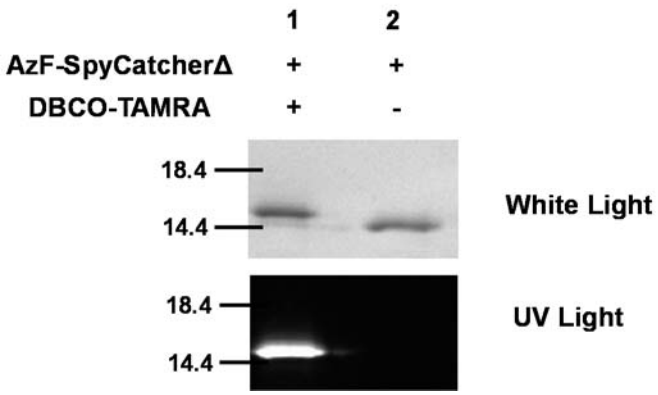

Figure S2. Unnatural amino acid AzF is successfully incorporated into SpyCatcherΔ. Purified AzF-SpyCatcherΔ (10 µM) was incubated with dye DBCO-TAMRA (500 µM, MW 937 Da, Click Chemistry Tools) in DPBS (pH 7.4) for 1 hr at room temperature. The mixture was then analyzed on a 12% SDS PAGE gel. The incorporation of each molecule of DBCO-TAMRA increases the molecular weight of the AzF-SpyCatcherΔ by ~1 kDa. Unreacted AzF-SpyCatcherΔ was used as the negative control. The gel was first imaged under UV light, and then stained with Coomassie blue dye and visualized under white light. The slight increase of band size in lane one and the appearance of fluorescent band in the same lane confirmed the successful incorporation of AzF in the SpyCatcherΔ.

1. Morizono, K, Bristol, G, Xie, YM, Kung, SK, and Chen, IS (2001). Antibody-directed targeting of retroviral vectors via cell surface antigens. *J Virol* **75**: 8016-8020.
2. Pariente, N, Morizono, K, Virk, MS, Petrigliano, FA, Reiter, RE, Lieberman, JR*, et al.* (2007). A novel dual-targeted lentiviral vector leads to specific transduction of prostate cancer bone Metastases In vivo after systemic administration. *Molecular therapy : the journal of the American Society of Gene Therapy* **15**: 1973-1981.
3. Munch, RC, Muhlebach, MD, Schaser, T, Kneissl, S, Jost, C, Pluckthun, A*, et al.* (2011). DARPins: an efficient targeting domain for lentiviral vectors. *Mol Ther* **19**: 686-693.
4. Steiner, D, Forrer, P, and Pluckthun, A (2008). Efficient selection of DARPins with sub-nanomolar affinities using SRP phage display. *J Mol Biol* **382**: 1211-1227.
